# Altered A-to-I RNA Editing in Human Embryogenesis

**DOI:** 10.1371/journal.pone.0041576

**Published:** 2012-07-31

**Authors:** Ronit Shtrichman, Igal Germanguz, Rachel Mandel, Anna Ziskind, Irit Nahor, Michal Safran, Sivan Osenberg, Ofra Sherf, Gideon Rechavi, Joseph Itskovitz-Eldor

**Affiliations:** 1 The Ruth and Bruce Rappaport Faculty of Medicine, Technion, Haifa, Israel; 2 Department of Obstetrics and Gynecology, Rambam Health Care Campus, Haifa, Israel; 3 Cancer Research Center, Chaim Sheba Medical Center, Tel Hashomer and Sackler School of Medicine, Tel Aviv University, Tel Aviv, Israel; University of Newcastle upon Tyne, United Kingdom

## Abstract

Post-transcriptional events play an important role in human development. The question arises as to whether Adenosine to Inosine RNA editing, catalyzed by the ADAR (Adenosine Deaminase acting on RNA) enzymes, differs in human embryogenesis and in adulthood. We tested the editing of various target genes in coding (FLNA, BLCAP, CYFIP2) and non-coding sequences at their *Alu* elements (BRCA1, CARD11, RBBP9, MDM4, FNACC), as well as the transcriptional levels of the ADAR1 enzymes. This analysis was performed on five fetal and adult human tissues: brain, heart, liver, kidney, and spleen, as well as on human embryonic stem cells (hESCs), which represent the blastocyst stage in early human development. Our results show substantially greater editing activity for most adult tissue samples relative to fetal ones, in six of the eight genes tested. To test the effect of reduced A-to-I RNA editing activity in early human development we used human embryonic stem cells (hESCs) as a model and tried to generate hESC clones that overexpress the ADAR1–p110 isoform. We were unable to achieve overexpression of ADAR1–p110 by either transfection or lentiviral infection, though we easily generated hESC clones that expressed the GFP transgene and overexpressed ADAR1-p110 in 293T cells and in primary human foreskin fibroblast (HFF) cells. Moreover, in contrast to the expected overexpression of ADAR1-p110 protein following its introduction into hESCs, the expression levels of this protein decreased dramatically 24–48 hr post infection. Similar results were obtained when we tried to overexpress ADAR1-p110 in pluripotent embryonal carcinoma cells. This suggests that ADAR1 protein is substantially regulated in undifferentiated pluripotent hESCs. Overall, our data suggest that A-to-I RNA editing plays a critical role during early human development.

## Introduction

RNA editing is a site-specific modification of an RNA sequence that yields a different product than that encoded by the DNA template. The most prominent RNA editing event in human cells is the substitution of adenosine to inosine (A-to-I), catalyzed by members of the double-stranded RNA-specific Adenosine Deaminase Acting on the RNA (ADAR) family of enzymes. Since inosines (I) in mRNA are recognized as guanosines (G) by the ribosome during the course of translation, RNA editing can lead to the formation of an altered protein if editing results in a codon exchange. Thus, RNA editing is an essential post-transcriptional mechanism for expanding the proteomic repertoire [1], [2]. Three separate ADAR gene family members, ADAR1, ADAR2, and ADAR3, were identified in humans and rodents [3], [4]. ADAR1-deficient mice were found to be embryonic lethal, while ADAR2 knockout mice appeared to develop normally but died during or soon after weaning [5], [6], [7]. Altered editing patterns were found to be associated with a number of human diseases including inflammation, epilepsy, depression, amyotrophic lateral sclerosis (ALS), and tumorigenesis [8], [9], [10], [11], [12]. In addition, RNA editing was shown to be involved in the regulation of nuclear retention [13] and human microRNA biogenesis [14], [15]. ADAR3 expression is restricted to the brain, yet no ADAR3 mediated editing has been reported, rendering ADAR3 function unknown. However, ADAR3 may act as an antagonist of the two other ADAR enzymes, either by competing on substrate binding or by forming non-functional hetrodimers with the other two enzymes [4].

Only a handful of known editing sites within coding sequence have been well characterized [16], [17]. Nevertheless, bioinformatic analyses have predicted A-to-I editing to be far more abundant than previously thought, apparently affecting thousands of human genes [18], [19], [20]. Most of the editing sites are located in non-coding regions, introns, and untranslated regions (UTRs). Editing sites are preferentially clustered in short interspersed elements (SINEs) such as *Alu* repetitive elements [18], [20]. The lesser amount of A-to-I substitutions in mice, rats, flies, and chickens than in humans is mainly due to the low representation of *Alu* repeats in those genomes [20], [21].

Literature describing RNA editing in human embryogenesis is limited. Low availability of human fetal samples and the complexity of measuring global RNA editing in various tissue samples are among the obstacles to such studies. In addition, the study of RNA editing role in stem cell biology is in a very early stage. Two recent publications have reported the involvement of ADAR enzymes and A-to-I editing in the regulation of adult stem cells, such as human neural progenitor cells [22] and mouse hematopoietic stem cells [23].

Edited RNA was recently shown to escape nuclear retention in undifferentiated hESCs, suggesting a specified role for non-coding edited RNA in hESCs [24]. hESCs are pluripotent cells that are derived from in vitro fertilized oocytes cultured to the blastocyst stage. These cells remain undifferentiated during prolonged propagation in vitro and retain a stable normal karyotype. hESCs can show true pluripotency and can potentially be induced toward differentiation, in vitro and in vivo, into all cell lineages [25]. Decreased editing levels of *Alu* sequences were recently observed during spontaneous differentiation of hESCs; and ADAR1 knockdown was shown to result in increased expression of genes involved in differentiation [26].

In the current study we analyzed the RNA editing levels of single sites at three coding genes: BLCAP, FLNA, and CYFIP2 [27], and of non-coding sites at the *Alu* elements of five genes: BRAC1, CARD11, RBBP9, MDM4, and FANCC. We compared RNA editing in samples derived from human fetal tissue and adult tissue, and assessed mRNA expression levels of ADAR enzymes. Simultaneously, we analyzed the editing efficiency of the same sites in hESCs. Finally, to further study the function of ADAR1 in early human embryogenesis we attempted to generate overexpression of ADAR1 in hESCs.

## Materials and Methods

### Cell and Tissue Samples

Ten human embryos aborted at 10–20 weeks gestation due to social reasons were obtained from women who signed consent forms that were approved by the Helsinki Committee for Genetic Experiments on Human Subjects at Rambam Health Care Campus, Haifa, Israel. Tissue samples were dissected within one hour post mortem and placed immediately in liquid nitrogen for subsequent storage at −80°C until RNA isolation. Adult tissue RNA was obtained from different sources. Brain, spleen, and liver samples were obtained from commercial sources: Clontech (Mountain View, CA, USA), Ichilov hospital (Tel Aviv, Israel), and Ambion (Austin, TX, USA) FirstChoice® Human Total RNA Survey Panel (Ambion RNA samples are comprised of pooled RNA from 3 donors). Adult heart and kidney RNA samples were obtained from commercial sources and isolated from biopsies. For adult tissue biopsies, written informed consent was obtained from each patient prior to surgery and approved by an institutional review board in accordance with guidelines for experiments on human subjects. Adult heart RNA was obtained from Clontech (pooled RNA from 24 male/female donors), Ambion (pooled RNA from 3 donors), and isolated RNA from a biopsy obtained during elective coronary artery bypass surgery (a 34 year- old male). Adult kidney RNA was obtained from Clontech (pooled RNA from 14 male/female donors), Ambion (pooled RNA from 3 donors), and isolated RNA from a biopsy of normal renal tissue obtained during elective surgery.

Human embryonic stem cell (hESC) lines H9.2 I6 and I3.2 were grown as described [25], [28], [29]. The embryonal carcinoma cell lines were obtained previously, and cultured in conditions similar to those of hESCs (NTERA2 cell line was obtained from the ATCC http://www.atcc.org, and the 2102 Ep cell line was a gift from Prof. P. Andrews, University of Sheffield, UK).

### RNA Purification and Analysis

Frozen tissue samples (∼1 mg) were homogenized in 1 ml TRizol reagent (Invitrogen, Carlsbad, CA, USA ), using Diax-100 Heidolph homogenizer (Heidolph, Germany). Total RNA was isolated from tissue samples and from hESC samples according to manufacturers' instructions. One µg RNA was subjected to SuperScript™ II Reverse Transcription (RT) reaction (Invitrogen) to generate cDNA. The cDNA samples were subjected to Quantitative Real-Time PCR analysis (AB, Foster City, CA, USA). Relative quantification (RQ) was performed in triplicate and normalized by the internal endogenous GAPDH expression. The reaction was performed using SybrGreen (ABI) with the following primers:

**ADAR1common** Fw: ACAGCCAAAGACACTCCCTCTC, Re: GGCTCAGCATGGCTATCTGG. **ADAR1-p110** Fw: GGCAGCCTCCGGGTG, Re: CTGTCTGTGCTCATAGCCTTGA. **ADAR1-p150** Fw: CGGGCAATGCCTCGC Re: AATGGATGGGTGTAGTATCCGC. **ADAR2** Fw**:** CCGCAGGTTTTAGCTGACG Re: CGGTCAGGTCACCAAACTTACC. **ADAR3** Fw: TTGGAAGGAGGCACCGACA, Re: CTTATTGGTTTCTCTGGGGCTG **GAPDH**: Fw: AGCCACATCATCGCTCAGACA, Re: GTACTCAGCGGCCAGCAT.

### Analysis of (A-to-I) RNA Editing

Editing quantification by primer extension combined with Sequenom analysis: RNA editing quantification was carried out using MALDI-TOF mass spectrometer (Sequenom, San Diego, CA) as described [30] for mutation detections. In brief, for each editing site, two specific primers flanking the editing site and one extension primer that bind an adjacent sequence to the editing site were designed using MassARRAY® assay design software (Sequenom). Primer sequences and editing site genomic localizations are listed in Table S1 (reference human genome- Mar. 2006 assembly, UCSC). Following amplification of the region of interest, a primer extension reaction was carried out. This reaction included sequence-specific hybridization and sequence-dependent termination that generated different products for the non-edited and edited cDNA fragments, each with its unique mass values. The editing level was determined by spotting the extension products onto silicon chips preloaded with proprietary matrix (SpectroChip; Sequenom) that were subsequently read by the MALDI-TOF mass spectrometer. Mann-Whitney statistical analysis was performed to identify significant differences in editing levels between samples.

### Transfection and Lentiviral Infection of ADAR1-p110 Isoform into hESCs

Plasmid construct: pCDNA3.1/HisC-ADAR1-p110 (a gift from Prof. C. Samuel, UCSB, CA) was used for the transfection experiments. For lentivirus infection, the ADAR1-p110 cDNA was excised from pCDNA3.1, using BamH1 and XhoI, and subcloned into the lentiviral construct PTK (a gift of Prof. G. Neufeld, Technion, Israel). Plasmid Transfection: 3 µg pCDNA3.1-p110 plasmid was transfected using Lipofectamine 2000 transfection agent (Invitrogen) into I3.2 and H9.2 hESCs and 293T cells. Following several days of G418 selection, 40 stable clones were mechanically selected, re-plated, and allowed to expand.

Virus construction and infections: for infection of hESCs, constructs harboring transgen, packaging (Gag-Pol), and VSVG (envelope) DNA (at a ratio of 10∶9∶1; 30 µg overall) were transfected using CaCl2/HBS (Na2HPO4; NaCl; Hepes) into 293 T cells, which were plated 24 hr before transfection, at a density of 2.5×10^6^ in a 10 cm plate, and supplemented with 25 µM chloroquine. The medium was switched 24 hr post transfection, and the supernatant containing the viruses collected 48–72 hr post transfection for either direct infection or freezing until infection.

One day prior to the infection, 10 µM Rho***-***associated protein kinase (ROCK) Inhibitor Y-27632 (Calbiochem, San Diego, CA, USA) was added to the hESC culture medium for 1 hr before collagenase treatment. After detachment from the MEF feeder layer using collagenase, the hESCs were treated with 0.5% trypsin and re-plated as single cells on fibronectin-covered 6-well dishes, at a density of ∼2.5×10^5^ cells per well, in MEF-conditioned medium (CM) supplemented with Rock inhibitor. Two days post transfection, supernatant from 293T cells was collected, filtered through a 0.45 μ filter and concentrated using Amicon (Millipore, Billerica, MA, USA) at 5000 g for 20 min. Concentrated viral supernatant was added to the hESCs CM and supplemented with ROCK inhibitor and 8 µg/ml Polybrene (Sigma-Aldrich, Rehovot, Israel). Twenty-four hours later, a second round of infection was conducted. Forty-eight hours post infection, hESCs were detached from fibronectin and either re-plated on MEF for further expansion or harvested for subsequent analysis.

### Western Blot

Protein was extracted from cells using RIPA lysis buffer (150 Mm NaCl, 1% NP-40, 0.5%deoxycholic acid, 1% SDS, and 50 mM TRIS pH 8). Extracted proteins were separated by migration on SDS Polyacrylamide Gel Electrophoresis (SDS PAGE), followed by transferring for 1 h in 300 mA onto a nitrocellulose membrane. The membrane was then blocked with 5% milk powder in TBST (50 mM Tris base, 150 mM NaCl, 0.1% Tween20), and incubated with anti ADAR1-p-110 primary antibody (#K188, generous gift from Prof. C. Samuel, UCSB, CA), overnight at 4°C. In the morning, the antibody residues were washed using TBST, and the membrane incubated with secondary goat anti–rabbit conjugated to horseradish peroxidase (HRP) antibody, diluted 1∶5000 in blocking solution for 1 hr. After washes with TBST, the reaction was performed using the ECL kit. Protein loading was verified using anti-actin Mab and secondary rabbit α-mouse antibody.

### PCR for Genomic DNA

Genomic DNA was extracted by the Wizard Genomic DNA purification kit (Promega, Madison, WI, USA) according to the manufacturer's instructions. PCR was performed using DreamTaq green master mix (Fermentas, Burlington, Canada) with the following primers: primers corresponding to the Rev Response Element (RRE) of the lentivirus construct: Fw-ACGGTACAGGCCAGACAATTA Re-GGTGCAAATGAGTTTTCCAGA; primers corresponding to a segment including part of the CMV promoter (left primer) and part of the ADAR1-p110 sequence (right primer):Fw: TACATCAATGGGCGTGGATA Re: GCATCCTCTCTCGCTTCTTG; and Genomic OCT4 segment: Fw: CCTTCCCTCCTTTACCCTACTCC Re: CCACCCCTGCTGCCTCTATT.

## Results

### Selection of Targets for Comparing Editing of Human Fetal Tissues Relative to Adult Tissues

To analyze editing levels of different fetal tissues we collected 10 human embryos of 10–20 weeks gestation, which were aborted due to social reasons. Fetal tissues were dissected and RNA was isolated from five tissues: brain, heart, kidney, liver, and spleen. Editing levels were measured using MALDI-TOF mass spectrometer, and compared to commercial RNA samples from respective human adult tissues.

In light of the large number of A-to-I editing sites in the human genome, and the lack of an affordable and validated high throughput method to analyze them, a panel of eight functional genes for which editing activity had been validated and calibrated [12], [31], was designed. The panel included three known editing sites within coding regions in which editing has been shown to alter coding sequences. The selected genes were bladder cancer associated protein (BLCAP), cytoplasmic familial mental retardation interacting protein 2 (CYFIP2), and filamin A (FLNA). Since most A-to-I editing was shown to occur within *Alu* elements in non-coding regions [18], five *Alu* repeat sequences containing transcripts were selected for investigation due to their relevance to cancer and development, and their capability to regulate cell growth, proliferation, and normal differentiation. The panel included retinoblastoma binding protein 9 (RBBP9), Caspase recruitment domain family 11 (CARD11), Fanconi anemia complementation group c (FANCC), double minute 4 (MDM4), and breast cancer 1 (BRCA1).

### Editing Level is Reduced in Fetal Tissues of Non coding *Alu* Elements

**Figure 1:**
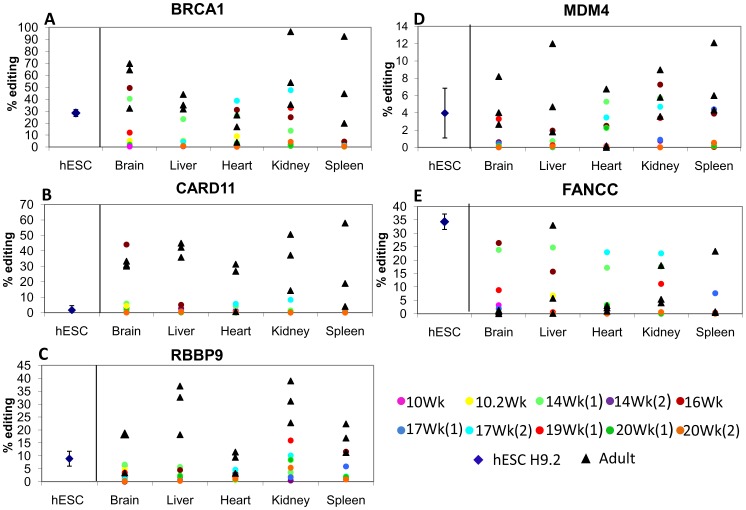
RNA editing activity of *Alu* elements is reduced in human fetal tissue samples relative to adult tissue samples. RNA editing levels were determined for samples of fetal tissues, adult tissues, and hESC lines H9.2 and I6 at passages 50–60. Mean levels of two independent measurements are presented, as assessed using the Sequenom Mass ARRAY compact analyzer. Editing level was significantly reduced for most fetal tissues in four genes tested: BRACA1 (A), CARD11 (B), RBBP9 (C), and MDM4 (D) (Mann-Whitney, P<0.05). FANCC (E) exhibited no difference between adult and fetal editing activity.

The highest level of RNA editing was detected at the BRCA1 gene. The tumor suppressor nuclear phosphor protein, BRCA1, is known as a breast and ovarian cancer susceptibility gene, presenting in 21%-40% of women with these types of cancer. The BRCA1 mutation can function as a predictive marker of response to chemotherapy [32]. In the current study, the BRCA1 sequence was highly edited in adult tissue samples, particularly in the kidney, spleen, and brain, with editing activity of 50–60% (Figure 1A). In contrast, editing of the BRCA1 sequence was reduced in fetal kidney and brain samples, reaching 10–20% in the kidney and hardly detectable, below 5%, in the spleen (Figure 1A). BRCA1 editing in adult liver and heart tissues showed lower levels, about 30% and 17% respectively; compared to under 5% in most liver fetal tissue samples (Figure 1A). In the fetal heart samples, a BRCA1 editing level of 12% was observed, similar to levels measured in adult heart. Statistically significant differences in editing between fetal and adult samples (Mann-Whitney test p<0.05) were observed for 4 of the 5 tissues tested (Table S2). BRCA1 editing in the hESC samples was ∼25%, which is within the range of fetal sample editing (Figure 1A).

CARD11 is a protein that participates in apoptosis signaling through protein-protein interactions [33]. For BRCA1, RNA editing levels were higher in adult than in fetal tissue samples, 20–40% and 10% respectively (Figure 1B). Differences in RNA editing between adult and fetal samples were statistically significant for the five tissues tested (Mann-Whitney test p<0.05; table S2). RNA editing in CARD11 of hESCs was very low (∼2%; Figure 1B). Interestingly, only one fetal brain sample (the 16 wk, shown as a dark red circle) exhibited a high editing level of ∼40%. Levels of RNA editing in FANCC and BRCA1, of 30–40%, were also exceptionally high for this brain sample, indicating an overall elevation of editing activity for this specific embryo brain.

The RBBP9 gene encodes a protein that is involved in the complex that binds to retinoblastoma (RB) tumor suppressor and regulates its activity as a monitor of apoptosis. RBBP9 is involved in malignant transformation of cells [34]. In the current study, RNA editing of RBBP9 was about 30% in adult liver and kidney samples, and 5% and below in fetal samples, except the kidney, in which RNA editing was higher than 10% (Figure 1C). Although editing levels of the adult heart, brain, and spleen samples were relatively low (10–20%; Figure 1C), they were still significantly above those of the corresponding fetal samples (Mann-Whitney test p<0.05; Table S2). RBBP9 editing in hESCs was ∼10% (Figure 1C).

MDM4 expression is elevated or overexpressed in 10–20% of over 800 diverse tumor types [35]. In the current study, overall RNA editing of the MDM4 gene was low (5–7%) in all tissues tested; maximal editing activity of ∼10% was detected in adult liver and spleen samples (Figure 1D). However, editing of MDM4 transcript was essentially undetectable in fetal brain, liver, and spleen tissues (Figure 1D), (Mann-Whitney test p<0.05 for the difference between adult and fetal tissues; Table S2). In contrast, fetal heart and kidney samples exhibited ∼5% editing activity, resembling their adult counterparts (Figure 1D). hESCs editing activity was similarly low and ranged between 2 and 6%.

FANCC is essential for protection against chromosome breakage, and is involved in protein complexes that functionally interact and inhibit the pro-apoptotic protein kinase PKR. Mutations in these genes cause increased binding of PKR to FANCC and increased PKR activation, leading to growth inhibition of hematopoietic progenitors and bone marrow failure in Fanconi anemia (FA) disease [36]. In the current study, RNA editing levels for the FANCC transcript were very low or almost undetectable (1–5%) in most samples, with some exceptions exhibiting 20–30% editing activity. RNA editing levels in fetal samples were highly variable, with some fetal samples showing 20–30% editing activity, while others exhibiting very low to undetectable levels (Figure 1E). hESC editing was relatively high (∼35%). These results suggest that a complex tissue- specific mechanism may be involved in FANCC transcript editing.

### Editing Level is Reduced in Coding Regions of Fetal Tissue Samples

**Figure 2:**
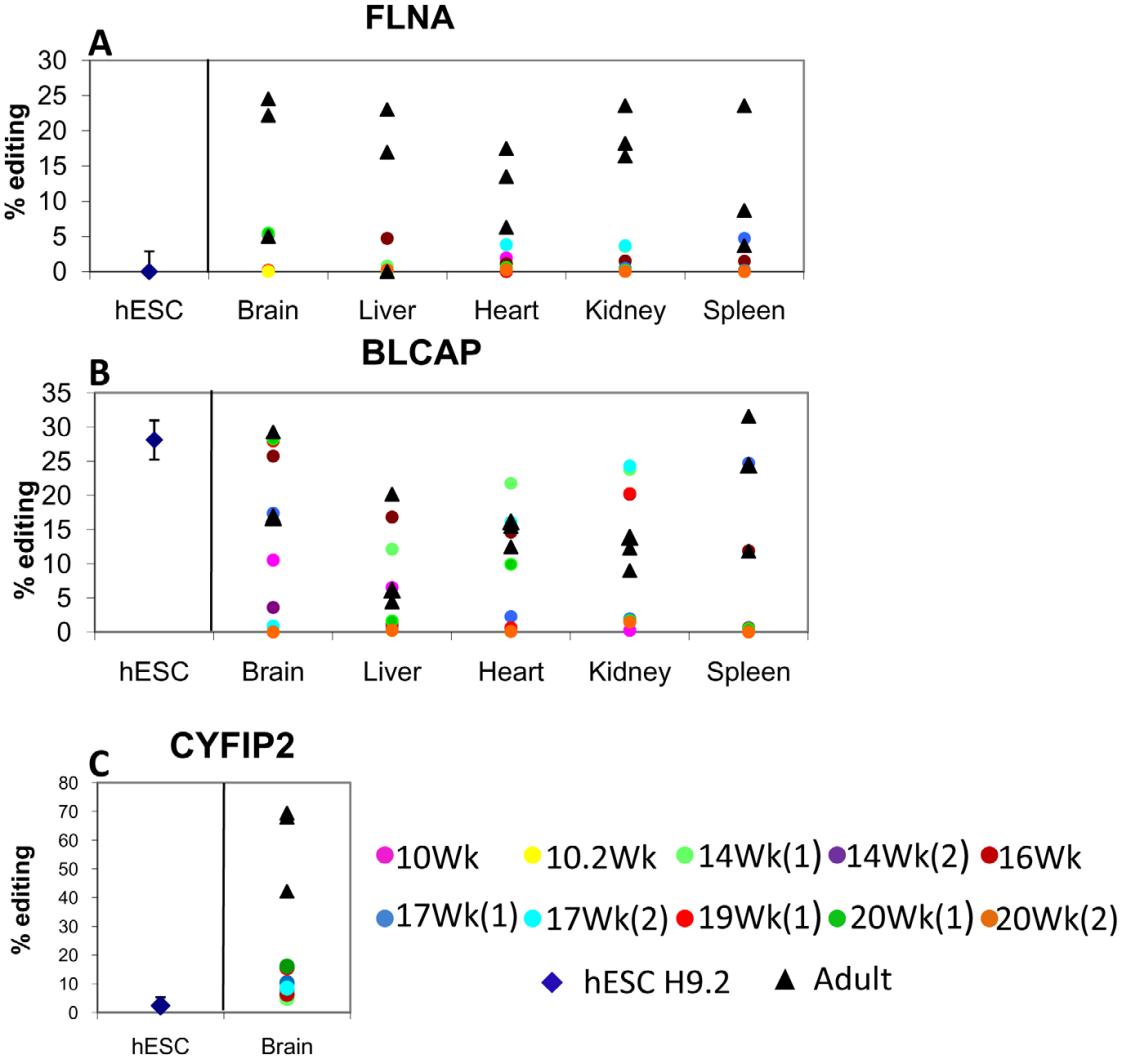
RNA editing activity of coding regions is reduced in human fetal tissue samples relative to adult tissue samples. RNA editing levels were determined for samples of fetal tissues, adult tissues, and hESC lines H9.2 and I6 at passages 50–60. Mean levels of two independent measurements are presented, as assessed using the Sequenom Mass ARRAY compact analyzer. Fetal editing of FLNA (A), BLCAP spleen (B), and CYFIP2 (C) was found to be significantly reduced (Mann-Whitney, P<0.05). No difference was found between fetal BLCAP (B) editing level of brain, liver, heart, and kidney compared to respective adult tissues.

FLNA**,** a ubiquitously expressed cell membrane anchoring protein, is involved in cell cytoskeleton element remodeling. RNA editing of FLNA results in Q to R substitution at amino acid 2341 of the human protein [27]. We detected editing in adult tissues of 10–20% (Figure 2A), compared to levels under 3%, which are essentially undetectable, in fetal tissues (Figure 2A; Mann-Whitney test p<0.05, Table S2). The FLNA substrate was not edited in hESCs.

BLCAP is expressed predominantly in the brain and B lymphocytes [37]. The few editing sites that have been identified in the BLCAP transcript occur in a tight cluster, containing several A-to-I substitutions (most of them in the intron and three in evolutionarily conserved sites in the coding sequence). We focused on the A-to-I editing event that results in the substitution of the second amino acid of BLACP from Y to C [27], [38]. RNA editing levels of BLCAP in adult brain and spleen were ∼20%, and in adult liver, heart, and kidney samples, 10–15% (Figure 2B). Fetal brain and kidney exhibited high variation in BLACP editing, with some samples showing over 20% editing activity, and others very low to undetectable levels of under 2% (Figure 2B). RNA editing of BLCAP was reduced in most fetal tissues tested compared to their adult counterparts; however, a statistically significant difference between adult and fetal tissues was observed only in the spleen (Mann-Whitney test p<0.05; Table S2). hESCs editing activity of BLCAP was relatively high and reached 25% (Figure 2B).

CYFIP2 is an evolutionarily conserved gene that is expressed predominantly in brain tissues, kidney, and white blood cells. CYFIP2 transcripts undergo A-to-I editing at amino acid 320, resulting in K to E substitution [27]. Here we show that RNA editing of CYFIP2 in the adult brain was significantly higher than in the fetal brain (Mann-Whitney test p<0.05; Table S2), reaching ∼70%, compared to 10% or less in fetal brain samples, (Figure 2C). For all other tissues tested (liver, kidney, heart, and spleen), CYFIP2 was either not expressed or RNA editing level was very low to undetectable (under 3%), in both fetal and adult samples (Data not shown).

In summary, most editing levels of known coding regions were significantly increased in adult tissue samples relative to fetal tissue and hESC samples. This is similar to the pattern for the *Alu* elements in four of the five non-coding regions tested (Figure 1). Overall, six of the eight genes tested exhibited statistically significant higher editing levels in most adult compared to fetal tissues (Mann-Whitney test p<0.05; Table S2). Therefore, it seems that in general, editing activity in adulthood is elevated relative to editing activity during embryogenesis. FANCC exhibited no significant difference (Table S2) between adult and fetal editing; and adult BLCAP editing activity was found to be significantly higher only in the spleen (Mann-Whitney test p<0.05; Table S2). These results suggest the involvement of a more complex tissue or gene specific editing mechanism.

### ADAR1 Transcript Expression, but not ADAR2 and ADAR3 Expression, is Reduced in Human Fetal Tissue Samples Relative to Adult Tissue Samples

**Figure 3:**
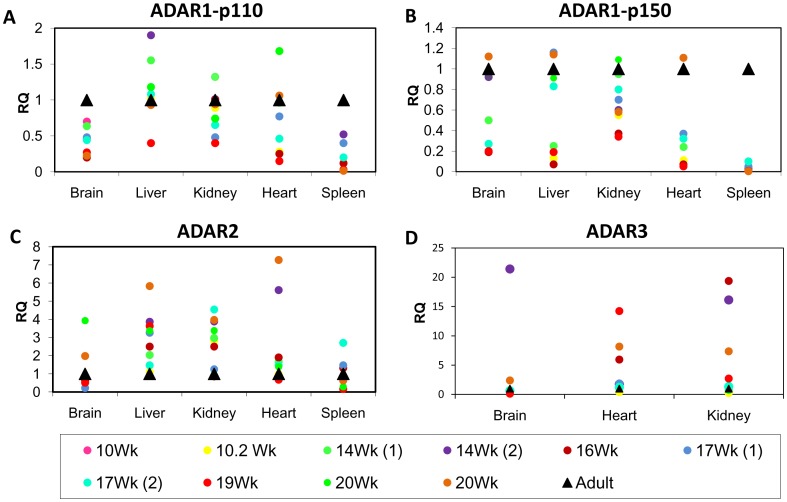
ADAR1 isoforms and ADAR2 mRNA expression levels in human fetal and adult tissue samples. 1 µg RNA, from samples obtained from fetal and adult tissues was reverse transcribed to generate cDNA. The mRNA expression level was determined using Quantitative Real Time PCR (QRT-PCR) analysis. The Y axis represents the relative ADAR expression levels of (A) ADAR1-p110 isoform, (B) ADAR1-p150 isoform, (C) ADAR2, and (D) ADAR3. The data plotted are representative of three independent experiments. It appears that ADAR1 transcript levels are mostly reduced, while ADAR2 transcript levels are mostly increased in human fetal tissues relative to respective adult tissues.

To investigate the mechanism responsible for the differences observed in RNA editing levels between fetal and adult tissues, we assessed the expression of ADARs, the editing enzymes, using quantitative real-time PCR. We found reduced transcript levels of ADAR1-p110 and p150 isoforms in fetal brain, kidney, heart, and spleen samples compared to levels in adult tissues (Figure 3A and B). The reduced expression levels of ADAR1 in most fetal tissues might explain the reduced RNA editing in these fetal samples. However, most fetal liver samples expressed higher levels of ADAR1-p110 compared with the adult liver sample (Figure 3A). Notably, the mRNA expression levels of ADAR1-p110 isoform were at least 10 fold higher than in the p150 isoform (data not shown).

ADAR2 transcript expression levels in adult brain and spleen tissues were equal, on average, to those of fetal tissues (Figure 3C). In contrast, lower ADAR2 expression level was observed in adult liver, kidney, and heart, compared to fetal tissue samples (Figure 3C).

We sought to examine whether the changes In ADAR2 expression affect the editing level of the glutamate receptor B (GluR-B) Q/R editing site, a well studied ADAR2 editing substrate [39]. The GluR-B Q/R site was completely edited, in both the adult and fetal tissues (Table S3) in which it was expressed, regardless of differences in the ADAR2 expression level.

The ADAR3 expression pattern was tissue- type dependent and not developmentally dependent. The ADAR3 transcript expression level in the brain was lower in most embryos than in adult samples (Figure 3D), with one embryo (14 wk(2), shown as a purple circle; Figure 3D) exhibiting an exceptionally high ADAR3 level. In contrast, the profile of heart and kidney expression was more complex, with most (but not all) embryos exhibiting increased ADAR3 transcript expression in the heart and kidney compared to adult samples (Figure 3D). Nevertheless, we note, that as shown before [4], ADAR3 was mostly expressed in the brain. In the other adult tissues tested, the ADAR3 level was very low (heart and kidney) or undetectable (spleen and liver). The relatively high levels of ADAR3 in fetal heart and kidney may indicate a novel role for ADAR3 in human embryogenesis.

### Efforts to Generate ADAR1-p110 Overexpression in hESCs

To study the mechanisms regulating reduced RNA editing and ADAR1 expression in fetal tissues and hESCs, we attempted to generate ADAR1 gain of function in hESCs. We chose the ADAR1-p110 isoform since this isoform was found to be reduced in most fetal tissue samples compared with respective adult tissue samples (Figure 3), and since editing of *Alu* elements in hESCs, as well as promiscuous editing of SINEs in mouse, were shown to be highly dependent on ADAR1 [26], [40].

With the intention of generating hESC ADAR1-p110 overexpressing clones, we transfected H9.2 hESCs with neomycine selectable plasmid, containing human ADAR1-p110 gene conjugated to N-terminal 6XHis-tag and Xpress epitope under the CMV promoter [41]. The transfected cells were subjected to antibiotic selection and 40 resistant clones were selected, expanded, and analyzed for ADAR1-p110 overexpression (OE). As a control, we transfected 293T cells with the same plasmid.

**Figure 4:**
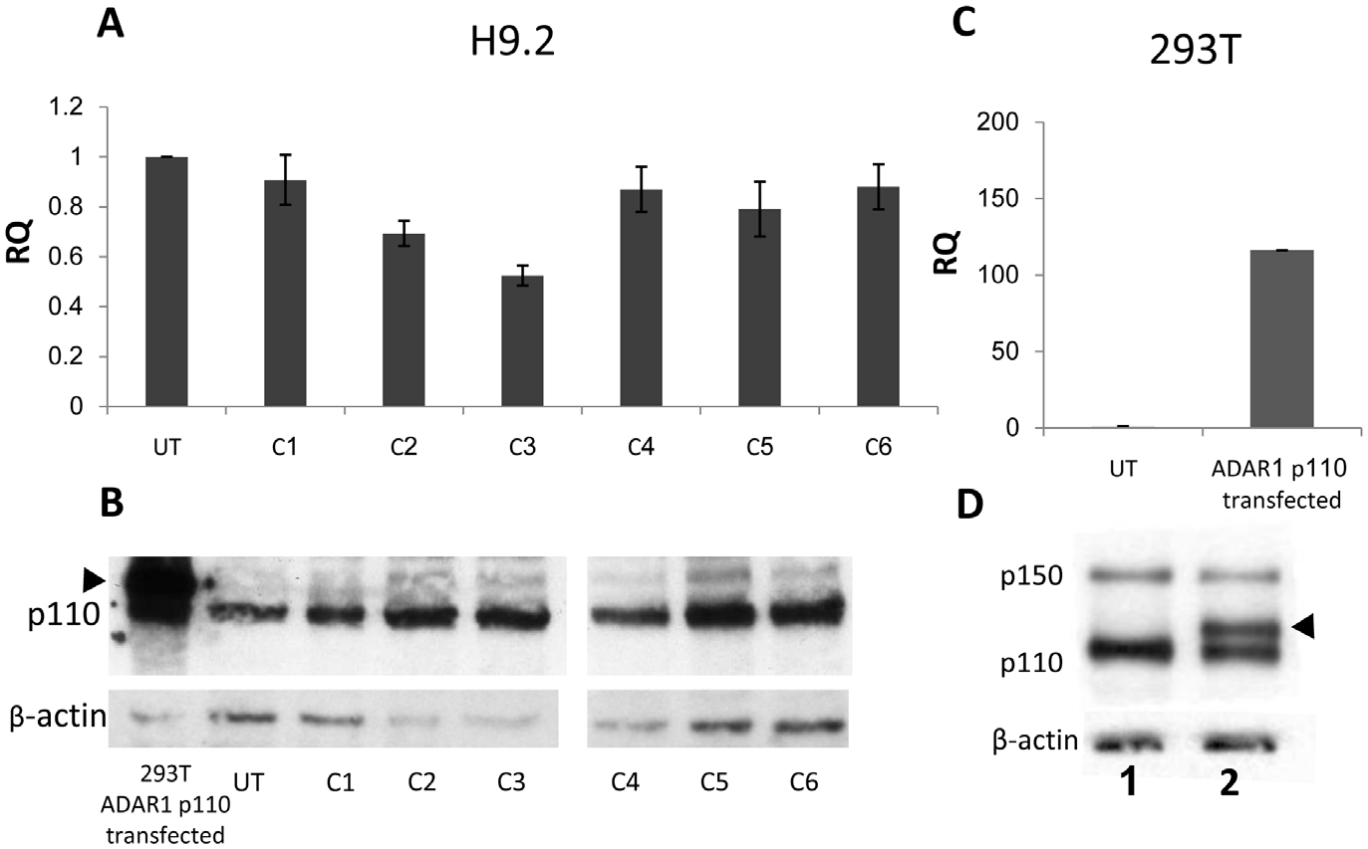
Transfection of ADAR1-p110 gene into hESC line H9.2. Attempts to generate stable hESC lines H9.2 overexpressing ADAR1-p110 failed: The pcDNA3.1/HisC plasmid, expressing ADAR1-p110 conjugated to His-tag and Xpress epitope at the amino end of the protein (which adds approximately 6 kDa to the protein) under the CMV promoter, was transfected into H9.2 hESCs. A similar plasmid was transfected into 293T cells for validation of plasmid functionality. H9.2 transfected cells were cultured on Neomycine (Neo) resistant Mouse Embryonic Fibroblast (MEF) feeder cells, and selection was performed with 50 µg/ml Neo for 10 days. Neo-resistant hESC clones were isolated and expanded. RNA and protein extracts were generated from 40 selected hESC clones and subjected to (A) QRT-PCR analysis and (B) Western Blot analysis. Representative data for 6 Neo-resistant hESC clones (C1–C6) are shown here relative to untransfected hESC H9.2 samples. For the Western Blot a positive control of 293T transfected cells, overexpressing ADAR1-p110 was added (left lane). Arrow head indicates the exogenous His-tag conjugated ADAR1-p110. No hESC Neo-resistant clone showed significant overexpression of ADAR1-p110 isoform following transfection and Neo-selection. 293T cells were harvested 4 days post transfection. RNA and protein were extracted from untransfected (UT) and transfected cells. ADAR1-p110 expression was analyzed by (C) QRT-PCR and (D) Western Blot analysis, lane 1– control untransfected cells, lane 2– transfected cells. Results show that ADAR1-p110 overexpression could be induced in 293T cells but not in selected hESC clones.

Of the ADAR1-p110 OE hESC clones generated, none overexpressed ADAR1-p110. In representative data for six selected hESC clones, none of the neomycin resistant clones exhibited increased mRNA (Figure 4A) or protein expression (Figure 4B) for ADAR1-p110 relative to untransfected H9.2 cells.

In contrast, we were easily able to generate extensive OE of ADAR1-p110 following transfection of 293T cells with the same vector. Transfected 293T cells showed ADAR1-p110 OE both by RNA expression (Figure 4C) and by protein expression (Figure 4D), indicating that the transfected vector is functional. The two bands of ADAR1-p110 (Figure 4D, lane 2) indicate the endogenous (lower bend) and the exogenous (upper bend; arrow head) His-tag conjugated ADAR1-p110 proteins that were expressed following transfection of 293T cells. Only the lower p110 band is visible in the untransfected 293T cells (Figure 4D, lane 1).

To eliminate the possibility that the ADAR1-p110 protein level was reduced in hESC neomycine resistant clones due to extended passages, we generated stable ADAR1-p110 OE under CMV promoter using lentivirus infection, and analyzed the protein level immediately after the infection. Lentivirus infection is a robust procedure for introducing exogenous genes into hESCs [42]. Infection efficiency is ∼50% in hESCs, and the exogenous gene integrates into the infected cell genome, while hESC transfection efficiency is ∼5%, using the lipofection method, which also requires long selection and expansion of the resistant clones.

**Figure 5:**
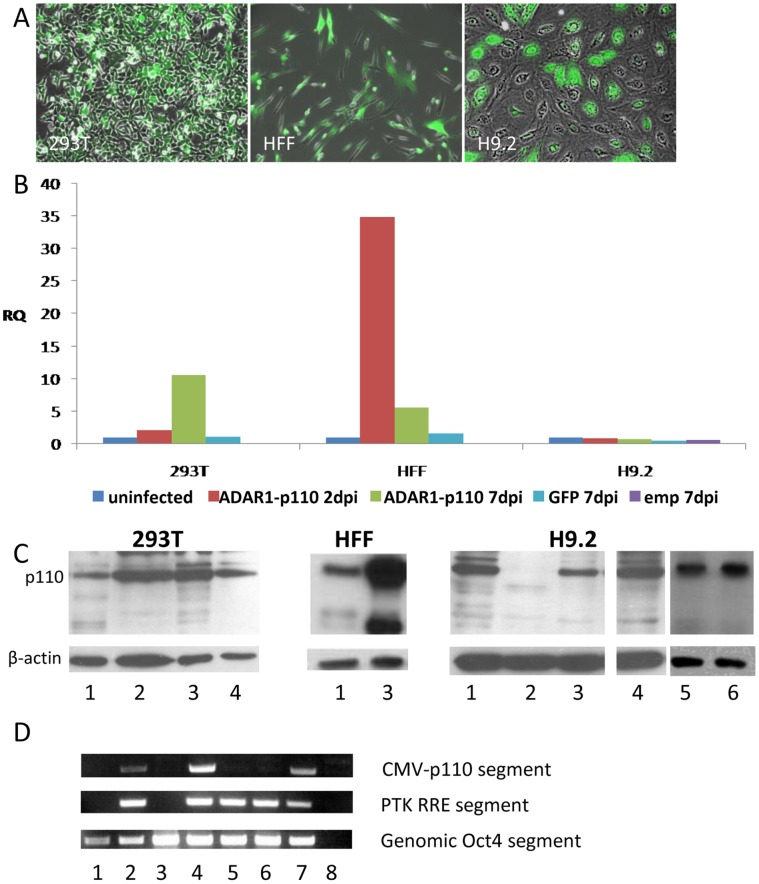
Lentivirus infection of ADAR1-p110 into the hESC line H9.2. ADAR1 overexpression could not be generated in H9.2 although the virus did integrate into the cells. H9.2 hESCs were infected with the lentivirus PTK vector harboring ADAR1-p110 gene under the CMV promoter. As a control, a similar plasmid harboring GFP under the CMV promoter or PTK with no insert (empty vector; emp) were used to infect H9.2 hESCs. Simultaneously, Human Foreskin Fibroblasts (HFF) and 293T cells were also infected with similar lentiviral vectors. (A) GFP-expressing cells demonstrated infection efficiency of ∼90% for 293T and HFF, and ∼50% for H9.2 hESCs. (B) mRNA expression levels of ADAR1-p110 transcript, following infection of H9.2, HFF, and 293T cells with lentivirus. Analysis was performed by QRT-PCR using specific primers, at time periods of two days post infection (2dpi) and seven days post infection (7dpi), relative to non-infected cells. (C) Western Blot analysis of ADAR1-p110 isoform protein level following infection of H9.2, HFF, and 293T cells. Infection was performed with the lentiviral vectors PTK-ADAR1-p110 and PTK-GFP. Protein was collected at 2dpi and 7dpi. Total protein levels were verified by β-Actin. Lanes: 1-uninfected cells, 2- PTK-ADAR1-p110 2dpi, 3- PTK-ADAR1-p110 7dpi, 4- PTK-GFP 7dpi, 5- PTK-emp 2dpi, 6- PTK-emp7dpi. (D) Analysis of lentivirus integration into H9.2, HFF, and 293T infected cell genome by PCR for genomic DNA. Primers were designed so that the left primer corresponds to the CMV promotor and the right to the ADAR1-p110 sequence. PCR for a genomic segment of OCT4 and the RRE element of the viral construct was performed as control. Lanes: 1–293T uninfected cells, 2- 293T, PTK-ADAR1-p110 7dpi, 3- H9.2 uninfected, 4- H9.2, PTK-ADAR1-p110 13dpi 5- H9.2, PTK-empty 7dpi, 6- HFF PTK-GFP 7 dpi, 7-HFF PTK-ADAR1-P110 7dpi 8- No template DNA - negative control.

The 293T cell line, and the primary cell human foreskin fibroblast (HFF), were infected simultaneously as control cells. Infection efficiency was estimated using a similar lentivirus construct harboring GFP under the CMV promoter. The infection rate was ∼50% for H9.2 cells and ∼90% for 293T and HFF cells (Figure 5A). Infected cells were harvested 24 hr, 48 hr, and 7days post infection, and ADAR1-p110 RNA (Figure 5B) and protein levels (Figure 5C) were examined. Lentivirus integration into the infected cell genome was verified by genomic PCR (Figure 5D). No ADAR1-p110 OE was detected at any time post infection in hESCs, while high levels of ADAR1-p110 mRNA (Figure 5B) and protein (Figure 5C) were demonstrated following infection of 293T and HFF cells with the PTK-ADAR1-p110 vector. Moreover, dramatic reduction of ADAR1-p110 protein was observed 24–48 hr post infection in hESCs (Figure 5C). The ADAR1 level was not reduced following infection of hESCs with the PTK empty vector or the PTK-GFP vector (Figure 5C). Seven days post infection the ADAR1-p110 protein level returned to the pre-infected H9.2 protein level (Figure 5C). Figure 5D demonstrates that the lentivirus vector integrated into the genome of the infected 293T, HFF, and hESCs. CMV-p110 segment insertion was detected even 13 days post ADAR1-p110 infection, refuting the possibility that ADAR1-p110 protein expression was reduced due to lack of survival of the PTK-ADAR1-p110 infected hESCs. These results suggest that ADAR1-p110 protein is not readily upregulated at the undifferentiated stage of hESCs due to an unknown mechanism.

### ADAR1 p110 Overexpression in Embryonal Carcinoma

**Figure 6:**
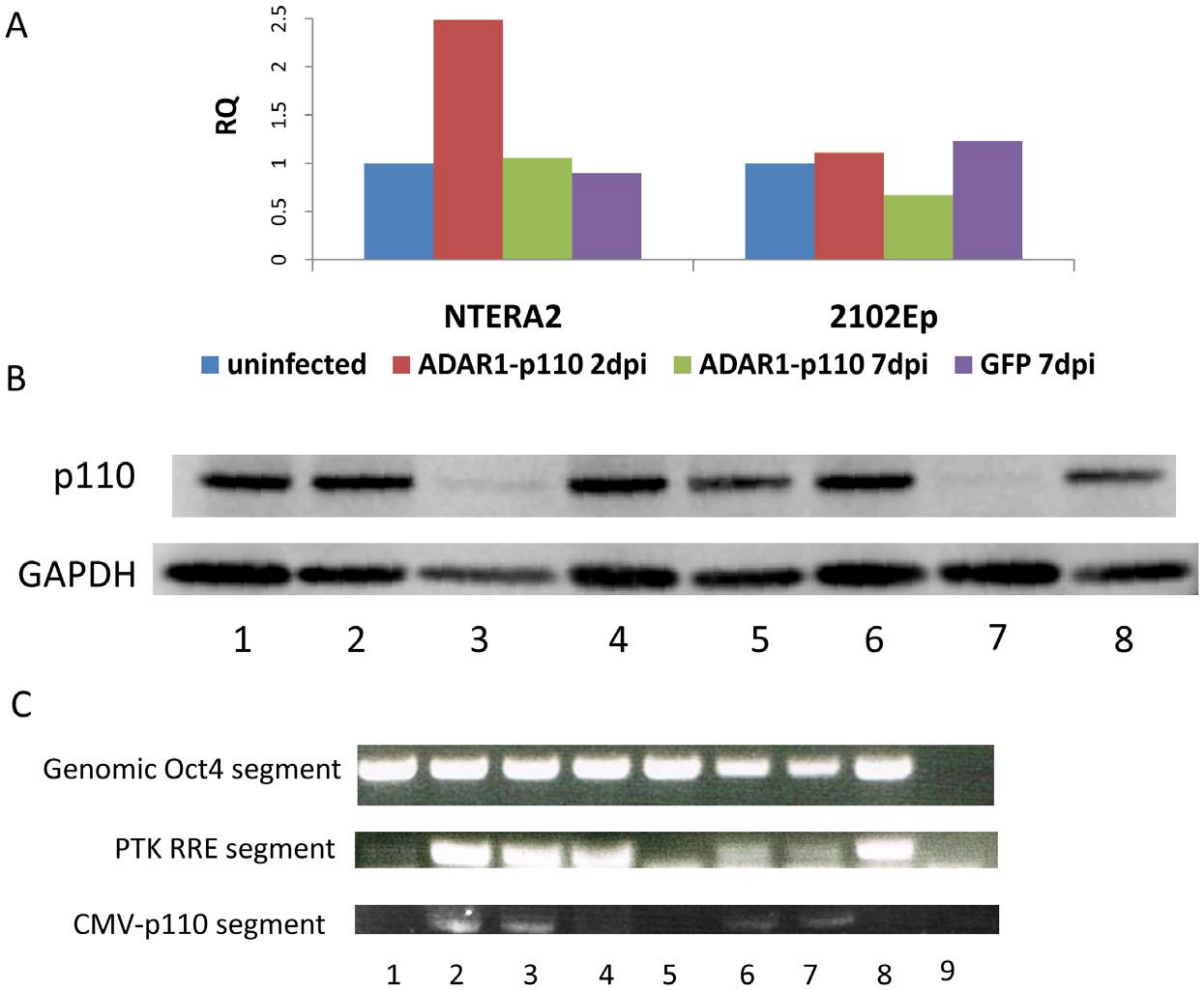
Lentivirus infection of ADAR1-p110 into the EC lines NTERA2 and 2102Ep. ADAR1-p110 overexpression could not be generated. THE EC lines NTERA2 and 2102Ep were infected with the lentivirus PTK vector harboring ADAR1-p110 gene under the CMV promoter. As a control, a similar plasmid harboring GFP under the CMV promoter was used. (A) mRNA expression levels of ADAR1-p110 transcript following infection. Analysis was done by QRT-PCR using specific primers, at 2dpi and 7dpi, relative to non-infected cells and normalized to GAPDH. (B) Western blot protein analysis. Lanes: 1-4- NETRA2, 5-8- 2102Ep; 1,5- uninfected, 2,6- ADAR1-p110 2dpi, 3,7- ADAR1-p110 7dpi, 4,8- 7dpi GFP infected. (C) Lentivirus integration into H9.2, HFF, and 293T infected cell genome verified by PCR for genomic DNA. Primers were designed so that the left primer corresponds to the CMV promotor and the right to the ADAR1-p110 sequence. PCR for a genomic segment of OCT4 and the RRE element of the viral construct was performed as control. Lanes: 1-4 NETRA2, 5-8 2102Ep; 1,5- uninfected 2,6- 15dpi, 3,7- 7dpi, 4,8- GFP infected 9- no template control.

Embryonal carcinoma (EC) cells are derived from teratocarcinoma benign tumors. EC cells are considered to be pluripotent cells since they exhibit both the signature expression of pluripotent markers and the ability to differentiate in-vivo into derivatives of the three germ layers [43]. Since ADAR1-p110 OE could not be achieved in hESCs (Figures 4 and 5), we tried to overexpress ADAR1-p110 in yet another pluripotent model. We used two well studied EC lines: NTERA2 and 2102Ep [44]. The EC cells were grown under hESC conditions and were infected with ADAR1-p110 in a procedure similar to that of hESCs. Results are presented in figure 6. NTERA2 exhibited minor elevation of ADAR1-p110 transcript shortly after infection (Figure 6A), while 2102Ep did not exhibit elevation in ADAR1-p110 at any time. For both EC clones, no increase in ADAR1-p110 protein level could be observed (Figure 6B). Genomic integration of the lentiviral derived exogenous ADAR1-p110 was verified (Figure 6C). In contrast to the reduction of p110 protein level post infection in hESCs (Figure 5C), no dramatic reduction in the p110 level was observed shortly after infection. However, at seven days post infection, both EC lines exhibited a decrease in the level of ADAR1-p110 protein (Figure 6B). These results further support our hypothesis that pluripotent stem cells are not easily susceptible to ADAR1-p110 overexpression.

## Discussion

Using RNA samples derived from multiple tissues, obtained from 10 pregnancies aborted at ages 10–20 weeks of gestation, as well as from hESCs, we were able to test RNA editing in early human embryogenesis. We compared the editing level of five embryonic tissues to respective adult tissues in five editing sites of non-coding sequence and three editing sites in coding regions. We found A-to-I RNA editing to be significantly higher in adult than fetal tissue of the same origin in two of three known coding regions, and in four of five non-coding regions (Figures 1 and 2).

Studies comparing normal and malignant human tissue samples, showed levels of RNA editing to be elevated or reduced in a gene specific manner. Editing levels of the *Alu* element sites in BRCA1 gene were increased, and those of the coding sequences in CYFIP2 and FLNA genes were reduced in glioblastoma multiforme brain tissue [12]. In contrast, another study did not find any difference in editing levels of similar target sites between urinary bladder cancer tissue samples and normal tissues [31]. However, bioinformatic analysis of A-to–I editing levels in various human cancers revealed general hypoediting in cancer tissues [12].

In the current study, the editing of CYFIP2 was ∼70% in the adult brain samples and ∼10% in the fetal brain samples (Figure 2C). Other tissues displayed only residual editing signals, as previously described [12], [27]. Paz et al showed that the editing of CYFIP2 in normal and malignant brain samples was ∼60% and ∼30%, respectively [12]. As a p53 inducible protein [45], CYFIP2 may be a pro-apoptotic gene. Thus, CYFIP2, like other editing targets, may be involved in an editing-dependent mechanism that regulates self renewal, differentiation, and apoptosis. Evidence is accumulating that malignant tumors are initiated and maintained by a population of tumor cells that share biological properties similar to those of normal stem cells. This “cancer stem cell” model is based on the observation that tumors arise from cells that can self renew and that have the potential to differentiate [46], [47]. We suggest that reduced RNA editing is important for extensive cell proliferation, which occurs in human embryogenesis, as well as in human tumorigenesis.

Two of the genes we tested did not show statistical difference in RNA editing between adult and fetal tissues. BLCAP editing was found to be significantly reduced in only one fetal tissue. This result concurs with previous results, which found no difference in the BLCAP editing level of lung and oral cavity cancer versus normal tissue [12]. Further, these results suggest the involvement of a more complex tissue or gene specific editing mechanism in these genes.

Knockout experiments in mice have demonstrated that RNA editing is crucial for normal development [5], [6], [7]. RNA editing was found to be primarily increased during animal development, with most studies focusing on rodent brain tissue. Hang and colleagues found that while the expression level of ADAR1 mRNA was constant during the development of rat brain, ADAR2 mRNA expression, as well as ADAR2 and 5HT_2C_R editing levels, were markedly increased during rat development [48]. In contrast, Jacobs and colleagues described a more dramatic induction of both ADAR1 and ADAR2 expression and increased editing activity in the mouse brain during development from embryogenesis into adulthood [49].

One study that examined the involvement of RNA editing in human brain development reported Q/R GluR-5 and GluR-6 editing sites to be developmentally up regulated, while GluR-B editing levels remained constant [50]. Similarly, we found the GluR-B Q/R site to be consistently fully edited in all human adult and fetal samples. Kawahara and colleagues further showed that ADAR1 and ADAR2 mRNA expression was slightly increased in adult human brain compared with fetal and neonatal brain samples [50]. These findings suggest a complex pattern of RNA editing regulation during human brain development [50]. We found that in all tissue types tested, mRNA expression levels of ADAR1 isoforms: p150 and p110, but not of ADAR2 and ADAR3, were elevated in adult samples relative to fetal ones (Figure 3). In contrast, lower or similar levels of ADAR2 and ADAR3 expression were observed in most adult compared to fetal tissue (Figure 3C and D). The increased expression of adult ADAR1, and particularly of its p110 isoform, with expression levels at least 10 fold higher than that of the p150 isoform, may explain the greater efficiency of RNA editing in most adult tissues, since it was suggested that promiscuous RNA hyper editing, particularly of SINEs, is highly dependent on ADAR1 [2], [40]. In contrast, for some genes no correlation between ADAR enzymes transcript expression and editing levels was observed. For example, though BLCAP is mainly edited by ADAR1 in both human and mouse [27], [40], no significant difference in BLCAP editing levels between adult and fetal samples was observed for most tissues. However, in other cases, insignificant differences between adult and fetal editing (Table S2) could be attributed at least in part, to an overlap between ADAR1 and ADAR2 editing substrate recognition [51]. This suggests the involvement of factors other than ADAR enzymes level in the regulation of RNA editing. The same conclusion was suggested by a team that used large scale RNA sequencing to test the editing efficiency of 28 sites, during mouse brain development [52]. That study reported gradually increasing global editing efficiency in the mouse brain, from embryogenesis to adulthood, despite constant expression of ADAR proteins [52]. In conclusion, it seems that global editing efficiency is increased during human development from embryogenesis to adulthood. Since ADAR enzyme mRNA expression level appears not directly correlated with this trend, probably another, yet unknown mechanism participates in the regulation of these alterations. Therefore, the differences in editing levels at various editing targets, as described herein and previously, suggest that RNA editing may play an important role in human development.

Originally isolated from the Inner Cell Mass (ICM) of human blastocysts, hESCs represent a very early stage of human development. These cells have virtually unlimited self-renewal capacity and developmental potential [25]. Of selected targets examined in hESCs, RNA editing was not detected in some, such as CARD11, FLNA, and CYFIP2, and was detected at low levels, of 10 to 30%, in others (Figures 1 and 2). From their recent study of ADAR1 expression level and editing activity of Lin28 and PAICS genes in hESC line H9, Chen et al. concluded that ADAR1 editing activity is robust in hESCs [24]. Furthermore, it was recently shown that editing of *Alu* sequences decreases during spontaneous differentiation of hESCs and that ADAR1 knockdown results in elevated expression of differentiation related genes [26].

To further study ADAR1 function in early development we attempted to generate ADAR1 gain of function in hESCs. We hypothesized that since editing and ADAR1 mRNA expression levels are reduced in early human development, and knockdown of ADAR1 affects the undifferentiated state of hESCs, ADAR1 could play an important role in controlling differentiation, self renewal, and pluripotency in hESCs. However, we were unable to achieve significant overexpression of ADAR1 mRNA or protein in the 40 antibiotic resistant clones we obtained from transfecting hESCs, even though these clones integrated into the plasmid containing the ADAR1-p110 isoform (Figure 4).

To eliminate the possibility of ADAR1 silencing during the passaging of hESC resistant clones, we used the lentivirus infection system to overexpress ADAR1-p110. As shown in Figure 5, we were able to achieve GFP expression in hESCs, as well as ADAR1 OE at the control cells, 293T, or primary HFFs. However, no ADAR1 OE could be generated in hESCs at any time post infection. Surprisingly, we observed a dramatic reduction in ADAR1-p110 protein at 24–48 hr post infection. The protein level returned to the pre-infection level at 7 days post infection, suggesting that ADAR1 protein is substantially regulated in undifferentiated hESCs. This contrasts with other cell types that easily overexpress this protein. We also tried to overexpress ADAR1-p110 in embryonal carcinoma, another pluripotent cell type, yet in both EC cell lines tested, no ADAR1-p110 protein OE was detected (Figure 6). As observed in hESCs, a reduction in ADAR1-p110 protein level was observed; however, unlike in hESCs, this reduction was observed 7 days post infection rather than shortly after infection. These data suggest that ADAR1-p110 protein expression is directly regulated in human pluripotent stem cells and that due to an unknown mechanism its overexpression is not readily achieved at the undifferentiated stage of hESCs and other pluripotent stem cells. Changes in mouse ESC properties were not reported following ADAR1 knockout [6], [7]. However, since RNA editing is much more prevalent in humans than in other mammals [21], the mechanisms regulating mouse and human ESCs may differ. More experiments, such as inducible ADAR1 overexpression, are needed to elucidate the involvement of ADAR1 protein in hESC regulation.

Overall, the differences observed in RNA editing between human embryonic and adult tissues, together with the unsuccessful ADAR1 overexpression in hESCs, indicate significant involvement of ADAR1 regulation during early human development.

## Supporting Information

Table S1 **Oligonucleotide list for the RNA editing analysis by SEQUENOME MassArray technology.** List of primer sequence and localization.
(DOCX)

Table S2 **Mann-Whitney test P values for differences in RNA editing levels between adult and fetal tissues.** Fetal and adult editing level were compared for statistical difference using Mann-Whitney Statistical analysis. Significant differences (p<0.05) were found in RNA editing between adult and fetal samples for 23 of 26 comparisons (shaded) of six genes (BRCA1, CARD11, RBBP9, MDM4, FLNA and CYFIP2). FANCC exhibited no significant difference between adult and fetal samples in RNA editing, and adult BLCAP editing was significantly higher only in the spleen.
(DOCX)

Table S3 **Editing % of the GluR-B Q/R site.** RNA editing levels were determined for samples of fetal tissues and adult tissues using the Sequenom Mass ARRAY compact analyzer. In fetal tissues GluR-B expression was observed in brain and most kidney and heart samples. In adult, GluR-B was not expressed in heart samples. For all tissues tested, GluR-B Q/R editing was essentially completely edited (almost 100%).
(DOCX)
