# Supplementary material for: Altered A-to-I RNA Editing in Human Embryogenesis
Source: PLoS One. 2012 Jul 31;7(7):e41576. doi: 10.1371/journal.pone.0041576 (PMC3409221; doi:10.1371/journal.pone.0041576)
Supplement: Table S1 — Oligonucleotide list for the RNA editing analysis by SEQUENOME MassArray technology. List of primer sequence and localization. (DOCX) [file pone.0041576.s001.docx]

| **Gene name** | **Forward primer for first PCR** | **Reverse primer for first PCR** | **Extension primer** | **Editing site Genomic localization** |
| --- | --- | --- | --- | --- |
| **BLCAP** | 5’ACGTTGGATGATTAGGTCGGTTCCTGCAGCACGTTGGATGATTAGGTCG | 5’CGTTGGATGAGGAGGACGGGCAGCAACGTTGGATGAGGAGGAC | 5’GCAGCCACTGGAGGCAA | chr20(-):35580986 |
| **CYFIP2** | 5’ACGTTGGATGTTTCGGCGACATGCAGATAG | 5’ACGTTGGATGACGTCCACTTGGACTTGTTC | 5’CTCTTCATAGTGAGCACTGGTCT | chr5(+):156669386 |
| **FLNA** | 5’ACGTTGGATGTTCGTGGTGCCTGTGGCTT | 5’ACGTTGGATGTGGGCGGTTTCTCTCGGTG | 5’TCACTGTTTCTAGCCTTC | chrX(-):153233144 |
| **RBBP9** | 5’ACGTTGGATGCCTTTTTTTGGCTGGGTGTGACGTTGGATGCCTTT TTT | 5’ACGTTGGATGGCCTCAAGCAATACAACCACACGTTG GATGGCCTCAA | 5’GGTGGCTCATGCCTGTA | chr20(-):18417182 |
| **CARD11** | 5’ACGTTGGATGAGATCACGCCACTGCACTC | 5’ACGTTGGATGCCAGGTCAAGCTTGCCTTTT | 5’CAGCCTGGTGACAGAGC | chr7(-):2942082 |
| **MDM4** | 5’ACGTTGGATGAAAAAAATCGTGGACCGGGC | 5’ACGTTGGATGACCTAGGTGATCTCCCAAAG | 5’AGTGGCTCACGCCTGTA | chr1(+):202787783 |
| **BRCA1** | 5’ACGTTGGATGTATTTTGAGATGGAGTCCAGC | 5’ACGTTGGATGGAAGTGCACGTTGCAGTGAG | 5’GTCGCCCAGGTTGGAGT | chr17(-):38523246 |
| **FANCC** | 5’ACGTTGGATGGGACTGGAGGAAGCACTAAT | 5’ACGTTGGATGTGAGCCTCAGAGGTTGAGAC | 5’AGAGATGGGGTTTCACC | chr9(-):96925560 |
| **GluR-B** | 5’ACGTTGGATGACCACACACCTCCAACAATG | 5’ACGTTGGATGTCTCTGGTTTTCCTTGGGTG | 5’GAAATATCGCATCCTTGC | Chr4(+):158257875 |

**Table S1: Oligonucleotide list for the RNA editing analysis by Sequenom**
