# Supplementary material for: Altered A-to-I RNA Editing in Human Embryogenesis
Source: PLoS One. 2012 Jul 31;7(7):e41576. doi: 10.1371/journal.pone.0041576 (PMC3409221; doi:10.1371/journal.pone.0041576)
Supplement: Table S2 — Mann-Whitney test P values for differences in RNA editing levels between adult and fetal tissues. Fetal and adult editing level were compared for statistical difference using Mann-Whitney Statistical analysis. Significant differences (p<0.05) were found in RNA editing between adult and fetal samples for 23 of 26 comparisons (shaded) of six genes (BRCA1, CARD11, RBBP9, MDM4, FLNA and CYFIP2). FANCC exhibited no significant difference between adult and fetal samples in RNA editing, and adult BLCAP editing was significantly higher only in the spleen. (DOCX) [file pone.0041576.s002.docx]

**Table S2: Mann-Whitney test P values for differences in RNA editing levels between adult and fetal tissues**

| **Coding regions** | | | **Non-coding regions** | | | | |  |
| --- | --- | --- | --- | --- | --- | --- | --- | --- |
| **Cyfip2** | **BLCAP** | **FLNA** | **MDM4** | **FANCC** | **RBBP9** | **CARD11** | **BRCA1** | **Gene/**  **Tissue** |
| 0.009 | 0.692 | \| 0.027 \| \| --- \| | \| 0.013 \| \| --- \| | \| 0.692 \| \| --- \| | 0.048 | 0.048 | 0.027 | **Brain** |
| - | 0.133 | 0.036 | 0.018 | 0.281 | 0.009 | 0.009 | 0.012 | **Liver** |
| - | 0.278 | 0.009 | 1 | 0.89 | 0.036 | 0.036 | 0.481 | **Heart** |
| - | 0.863 | 0.009 | 0.1 | 0.375 | 0.009 | 0.009 | 0.018 | **Kidney** |
| - | 0.048 | 0.033 | 0.033 | 0.095 | 0.033 | 0.016 | 0.016 | **Spleen** |
