# Supplementary material for: Altered A-to-I RNA Editing in Human Embryogenesis
Source: PLoS One. 2012 Jul 31;7(7):e41576. doi: 10.1371/journal.pone.0041576 (PMC3409221; doi:10.1371/journal.pone.0041576)
Supplement: Table S3 — Editing % of the GluR-B Q/R site. RNA editing levels were determined for samples of fetal tissues and adult tissues using the Sequenom Mass ARRAY compact analyzer. In fetal tissues GluR-B expression was observed in brain and most kidney and heart samples. In adult, GluR-B was not expressed in heart samples. For all tissues tested, GluR-B Q/R editing was essentially completely edited (almost 100%). (DOCX) [file pone.0041576.s003.docx]

**Table S3: Editing % of the GluR-B Q/R site**

|  | **10.2 Wk** | **14Wk (1)** | **14Wk (2)** | **16Wk** | **17Wk** | **19Wk** | **20Wk** | **Adult1** | **Adult2** | **Adult3** |
| --- | --- | --- | --- | --- | --- | --- | --- | --- | --- | --- |
| **Brain** | 98.89637 | 99.46008 | 99.76955 | 99.37085 | 98.64386 | 99.61747 | 99.92256 | 99.7473 | 99.17953 | 98.72613 |
| **Heart** | 98.51577 | 100 | - | 99.91998 | 99.89707 | 99.70953 | 99.59304 | - | - | - |
| **Kidney** | 98.25095 | 99.19405 | 96.67554 | - | 99.20405 | 98.87625 | 99.21962 | 99.53943 | 99.04392 | - |
